# Longitudinal Electroencephalography Analysis in Subacute Stroke Patients During Intervention of Brain–Computer Interface With Exoskeleton Feedback

**DOI:** 10.3389/fnins.2020.00809

**Published:** 2020-08-14

**Authors:** Shugeng Chen, Lei Cao, Xiaokang Shu, Hewei Wang, Li Ding, Shui-Hua Wang, Jie Jia

**Affiliations:** ^1^Department of Rehabilitation Medicine, Huashan Hospital, Fudan University, Shanghai, China; ^2^Department of Computer Science and Technology, Shanghai Maritime University, Shanghai, China; ^3^School of Mechanical Engineering, Shanghai Jiao Tong University, Shanghai, China; ^4^School of Architecture Building and Civil Engineering, Loughborough University, Loughborough, United Kingdom; ^5^School of Mathematics and Actuarial Science, University of Leicester, Leicester, United Kingdom; ^6^National Clinical Research Center for Aging and Medicine, Huashan Hospital, Fudan University, Shanghai, China

**Keywords:** BCI performance, event-related desynchronization, motor recovery, longitudinal change, stroke

## Abstract

**Background:**

Brain–computer interface (BCI) has been regarded as a newly developing intervention in promoting motor recovery in stroke survivors. Several studies have been performed in chronic stroke to explore its clinical and subclinical efficacy. However, evidence in subacute stroke was poor, and the longitudinal sensorimotor rhythm changes in subacute stroke after BCI with exoskeleton feedback were still unclear.

**Materials and Methods:**

Fourteen stroke patients in subacute stage were recruited and randomly allocated to BCI group (*n* = 7) and the control group (*n* = 7). Brain–computer interface training with exoskeleton feedback was applied in the BCI group three times a week for 4 weeks. The Fugl–Meyer Assessment of Upper Extremity (FMA-UE) scale was used to assess motor function improvement. Brain–computer interface performance was calculated across the 12-time interventions. Sensorimotor rhythm changes were explored by event-related desynchronization (ERD) changes and topographies.

**Results:**

After 1 month BCI intervention, both the BCI group (*p* = 0.032) and the control group (*p* = 0.048) improved in FMA-UE scores. The BCI group (12.77%) showed larger percentage of improvement than the control group (7.14%), and more patients obtained good motor recovery in the BCI group (57.1%) than did the control group (28.6%). Patients with good recovery showed relatively higher online BCI performance, which were greater than 70%. And they showed a continuous improvement in offline BCI performance and obtained a highest value in the last six sessions of interventions during BCI training. However, patients with poor recovery reached a platform in the first six sessions of interventions and did not improve any more or even showed a decrease. In sensorimotor rhythm, patients with good recovery showed an enhanced ERD along with time change. Topographies showed that the ipsilesional hemisphere presented stronger activations after BCI intervention.

**Conclusion:**

Brain–computer interface training with exoskeleton feedback was feasible in subacute stroke patients. Brain–computer interface performance can be an index to evaluate the efficacy of BCI intervention. Patients who presented increasingly stronger or continuously strong activations (ERD) may obtain better motor recovery.

## Introduction

Brain–computer interface (BCI) is increasingly developing in the neurological treatment, especially in stroke rehabilitation (López-Larraz et al., 2018). It is an intervention focused on the central nerve system, which played a role in both treatment and assessment. Recently, a meta-analysis (Cervera et al., 2018) has revealed its clinical efficacy in stroke motor rehabilitation. More and more studies have been performed to verify the positive effects in motor recovery by BCI training and to explore its possible mechanism as for promoting related cortical plasticity.

Brain–computer interface intervention has been applied to train stroke patients both in chronic and subacute stages for motor rehabilitation. Some scholars focused on chronic stroke. Ander et al. (Ramos-Murguialday et al., 2013) first reported a significant improvement in upper-limb motor function of the chronic stroke patients after 8 weeks in a randomized controlled trial. Leeb et al. (2016) also showed motor recovery through 10 sessions’ BCI training. Ang et al. (2014) applied 12-session BCI training in chronic stroke and reported motor improvements.

Literature reported that stroke patients might obtain more motor recovery in subacute than chronic stage (Buma et al., 2013). It suggested the possibility and value to add BCI intervention in subacute stage. As a result, studies of BCI intervention in subacute stroke were performed by several other scholars. Li et al. (2014) applied 24 sessions’ BCI training with functional electrical stimulation (FES) feedback in stroke patients of subacute stage and reported an improvement in hand function by the Action Research Arm Test. Pichiorri et al. (2015) reported an improvement in the Fugl–Meyer Assessment of Upper Extremity (FMA-UE) after 12-session BCI training. Mihara et al. (2013) applied six sessions’ BCI training and found improvement in the hand/finger subscale of FMA-UE. All above studies suggested the feasibility and efficacy to apply BCI intervention in both chronic and subacute stroke to promote motor recovery.

Brain–computer interface performance is a parameter used to judge the interaction effects when applying BCI training. Tam et al. (2011) tried to improve BCI performance to make it more user-friendly and reliable for stroke rehabilitation. Higher BCI performance was reported to be along with good motor recovery in stroke patients. Li et al. (2014) reported that BCI performance improved as the intervention times increased and the patients obtained motor recovery. Tung et al. (2013) reported that the number of sessions correlated with the change in the FMA scores. Brain–computer interface performance may be used to evaluate the applicability of BCI intervention for stroke individuals. Prasad et al. (2009) showed a range of 60–75% of online BCI performance in chronic stroke patients, which suggested the feasibility of BCI training in neurorehabilitation.

Sensorimotor rhythm changes were commonly explored by many BCI studies. Event-related desynchronization (ERD) (Pfurtscheller, 1979) was calculated from the electroencephalography (EEG) data to describe sensorimotor rhythm changes. It also stands for the cortical activities of the stroke patients during motor tasks (Pfurtscheller, 1999; Takemi et al., 2013; Kaiser et al., 2014). Stronger ERD was reported to present in the sensorimotor cortex of patients with good motor function (Bartur et al., 2019), and the location of ERD became focused on sensorimotor cortex after rehabilitation training (Tam et al., 2011; Liu et al., 2014). The longitudinal changes of cortical activities during a long-term BCI intervention are of great importance to show the time-varying effects. However, the longitudinal sensorimotor rhythm changes of patients with different levels of motor recovery under BCI intervention in subacute stroke are unclear. In subacute stage, the cortical activities varied quickly along time. During BCI training, it is valuable to clarify the longitudinal cortical activities, which may help explore the mechanism of BCI intervention.

The application of BCI intervention in subacute stroke patients could be useful in motor rehabilitation. The aim of this study was to explore the characteristics of BCI performance and longitudinal sensorimotor rhythm changes in subacute stroke patients. We hypothesized that the continuous results of BCI performance could be used to evaluate the clinical effects of motor recovery. Patients presented different longitudinal sensorimotor rhythm changes with different levels of motor recovery.

## Materials and Methods

### Research Subjects

Patients were recruited from the Department of Rehabilitation Medicine of Huashan Hospital affiliated to Fudan University. Inclusion criteria for the study were as follows: (1) unilateral subcortical stroke (ischemia or hemorrhage) diagnosed by computer tomography or magnetic resonance imaging (MRI); (2) first onset of stroke; (3) age between 25 and 75 years; (4) the onset was more than 4 weeks and less than 6 months; (5) the level of cognitive impairment: Mini-Mental State Examination score >25; (6) and being able to sit in a chair for at least 1 h. Exclusion criteria were as follows: (1) patients with previous history of epilepsy; severe failure of vital organs such as heart, lung, liver, and kidney; uncontrollable hypertension; arrhythmia; severe coronary heart disease; and diabetes complications; (2) unilateral neglect or vision problems; (3) allergic to conductive paste; (4) received other non-invasive brain stimulation interventions during the study period; and (5) cannot complete basic treatment. Fourteen subacute stroke patients were enrolled in the study and randomly allocated to BCI group (*n* = 7) and the control group (*n* = 7). Baseline demographic data and clinical characteristics of patients were presented in Table 1. This study was approved by the Ethics Committee of Huashan Hospital (KY2017-005) and performed according to the Declaration of Helsinki. All the patients signed the informed consent.

**TABLE 1:** The demographic and baseline clinical characteristics of the subjects.

	Sex	Age (years)	AL	TI	TSI (m)	SI	FMA-UE
**BCI group**							
OME1	M	31	R	I	5	L, basal ganglia	36
OME2	M	40	L	H	4	R, basal ganglia	30
OME3	M	42	R	H	1	L, basal ganglia	50
OME4	M	47	R	I	1	L, paracele	37
OME5	M	36	R	I	3	L, basal ganglia	28
OME6	M	30	R	I	5	L, paracele, basal ganglia	25
OME7	M	65	L	I	3	R, brainstem	13
Mean (*SD*)	–	41.6 (12.0)	–	–	3.1 (1.7)	–	31.3 (11.5)
**Control group**							
CG1	F	72	R	I	1	L, paracele	19
CG2	M	37	R	H	4	L, basal ganglia	28
CG3	F	43	L	I	3	R, basal ganglia	29
CG4	M	64	R	I	4.5	L, paracele, corona radiata	26
CG5	M	47	R	H	2	L, basal ganglia	28
CG6	M	64	R	I	6	L, brainstem, basal ganglia, paracele	42
CG7	M	42	L	I	4	R, basal ganglia	54
Mean (*SD*)	–	52.0 (11.1)	–	–	3.9 (1.5)	–	32.3 (11.8)
*p*	–	0.13	–	–	0.70	–	0.87

*AL, affected limb; TI, type of injury; TSI, time since injury; SI, site of injury; M, male; F, female; R, right; L, left; I, ischemia; H, hemorrhage; FMA-UE, the Fugl–Meyer Assessment of Upper Extremity; SD, standard deviation; p, results of t-test between groups.*

### Experimental Procedure

#### Group Allocation

In addition to the BCI intervention, the other parts of the basic treatment were maintained consistent in the two groups (BCI group and control group). Basic treatment included the following: (1) drug: following the rehabilitation physician’s advice; (2) routine rehabilitation therapy: physical therapy (20 min, five times a week), low-frequency electrical stimulation (20 min, five times a week), occupational therapy (20 min, five times a week).

The BCI intervention was three sessions a week lasting 1 month, with a total of 12 sessions. To keep the consistency of rehabilitation with the experimental group, the control group was instructed with the same hand motor imagery training tasks. All patients in the control group were instructed to attempt motion of wrist extension by the same therapist. The tasks were combined with three sets, 30 motions for one set. And the instructions were maintained the same as it were in the BCI group. Patients in the control group did not use the Omega device, and they were blind to what the patients did in the BCI group.

#### Protocol for BCI Intervention

**FIGURE 1:**
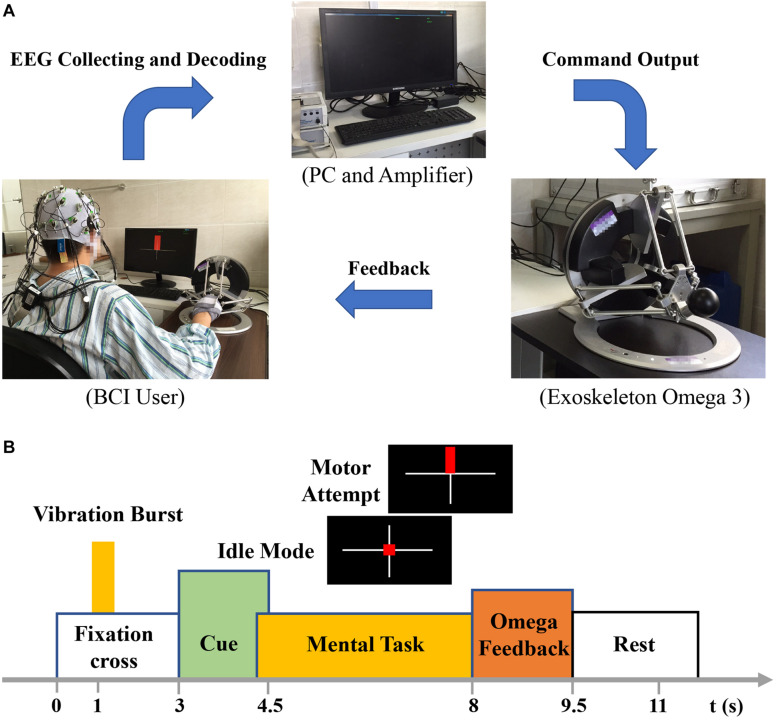
**(A)** Overview of the BCI system principle. **(B)** Experimental setup of one trial.

Figure 1A shows the overview of the BCI intervention system principle. Brain–computer interface training was conducted in a quiet room. The Omega force feedback device was placed on the table and controlled by the BCI system. The patients sat in front of a computer screen with their affected hands fixed on the Omega device. Electroencephalography were collected using 32 channels consisting of Ag/AgCl electrode of EEG cap (actiCAP; Brain Products, Gilching, Germany) according to the configuration of 10–20 International System (Klem et al., 1999). The signal is amplified by the amplifier (Brain Products). The reference electrode was located in the right mastoid process, and the ground electrode was located in the forehead. The electrode impedance was kept below 5 kΩ. The original EEG signals was recorded at a sampling rate of 200 Hz and filtered by a bandpass filter between 1 and 100 Hz.

There was approximately 11 s for one trial, 30 trials as a set with a total of three sets for one-session BCI training. Patients in the process of BCI training were required to attempt motion of wrist extension as far as possible but not to have compensatory movements (e.g., to move the head and shoulders, etc.). When the BCI system correctly recognized the intention of the patients’ motor attempt, it would output command and manipulate the Omega force feedback device and drive the patients’ affected hands to complete the wrist extension motion. When the patients’ motion intention was not successfully recognized, the Omega device would not produce any movement.

Figure 1B shows the experimental setup of one trial. During one trial, there was a white cross presenting on the center of the screen from 0 to 3 s. The patients kept still and rest. After the task began 1 s, vibrations appeared on the Omega device to give tips for the patients. And then a red square or a red rectangle appeared. The red square represented a static task, whereas a red rectangle represented the motor task. When the patients were performing motor task, they were required to maintain the motion as far as they could until the white cross disappeared. When there was a static task, patients just rested and did nothing. The rest time interval was adopted randomly in order to prevent the patients’ adaptability in the training process. There were rest intervals between each set (totally three sets for 1 day’s BCI training) generally for 1 min, depending on the patients’ status.

### BCI Performance Calculation

Brain–computer interface performance was evaluated between the task and idle states. The idle state was defined at [−4, −1] s prior to task cues and the task state was defined at [1, 4] s post-task cues. Common spatial pattern (CSP) was used for feature extraction of EEG data (Blankertz et al., 2006). Electroencephalography data were filtered by the band from alpha–beta frequency (8–30 Hz).

For online BCI performance, all 31 channels (FP1, FZ, F3, F7, FT9, FC5, FC1, C3, T7, TP9, CP5, CP1, PZ, P3, P7, O1, O2, P4, P8, TP10, CP6, CP2, CZ, C4, T8, FT10, FC6, FC2, F4, F8, FP2) were used for calculation. In each online test, the first run of 30 times of the task state and static state were used for training the classifier. In the second run of tests, at the end of each task, single-trial classification was performed with 31 channels of EEG signals. In order to better adapt to the nonstationarity of EEG signals, supervised online adaptation was implemented in the online decoding. In the second online feedback training, the CSP filter and linear discriminant analysis classifier were retrained after each test using the previous 30 test results.

For offline BCI performance calculation, all 31 and 7 channels (FC1, FC2, C3, CZ, C4, CP1, and CP2) were selected for data analysis. The first and last three rows of the CSP transformation matrix could be used for maximizing the difference of two groups of data. And then the transformed features were trained by random forest classifier. The 90 trials were divided into three pieces. Thus, a 3-fold cross-validation was applied to pick an optimal classifier.

### EEG Processing

Electroencephalography data from 31 channels were used in processing. The power spectrum of channels C3 and C4 was computed at the frequency of 8–30 Hz to identify ERD on motor tasks of the affected hands. Time–frequency distributions of EEG trials were estimated using a windowed Fourier transform (Peng et al., 2019) with a fixed 200 ms Hanning window. Windowed Fourier transform yielded, for each trial, a complex time–frequency estimate F(t,f) at each time–frequency point (t,f), extending from −2,000 to 6,000 ms (in steps of 5ms) in the time domain, and from 1 to 30 Hz (in steps of 1Hz) in the frequency domain. Power spectrum (P), P(t,f) = |F(t,f)|^2^, was obtained. The percentage of relative power change was calculated to obtain the ERD with respect to a resting-state baseline ([−2, 0] s) before the red triangle cue. The interest time was set at [1, 4] s after the cue, during which the patient was performing the motor tasks of wrist extension. The power spectrum of interest in the period after the event is given by *A*, whereas that of the preceding baseline period is given by *R*. Event-related desynchronization/event-related synchronization (ERS) (E) was calculated according to Eq. (1):

(1) $$E=\frac{A-R}{R}\times 100\%$$

Under this definition, ERD was expressed as a negative value, and ERS was a positive value. The topographies were drawn with an interest time of [1.4, 1.6] s after the task onset, with respect to a resting-state baseline ([−2, 0] s).

### Statistical Analysis

Analyses were conducted using SPSS version 23.0 (IBM Inc., Chicago, IL, United States). Continuous variables are presented as the mean ± standard deviation. A *t*-test was performed to compare the difference of age, time since injury, and baseline FMA-UE between the BCI group and the control group. Two-way repeated-measures analysis of variance (ANOVA) was performed for the FMA-UE with time as the within-subject factor (i.e., before and after therapy) and group as the between-subject factor (i.e., BCI and control groups). If a significant interaction was identified through two-way repeated measures ANOVA, then a paired *t*-test was adopted for *post hoc* analysis to compare FMA-UE score before and after BCI intervention. A *t*-test was conducted to compare the effects between groups. Two-way repeated-measures ANOVA was performed for the ERD with time (i.e., before and after therapy) and channel (i.e., C3 and C4) as the within-subject factors. A paired *t*-test was applied for *post hoc* analysis to compare the ERD before and after BCI intervention in the BCI group. *p* < 0.05 (two-sided) was considered to indicate a significant result.

## Results

### BCI Performance

**FIGURE 2:**
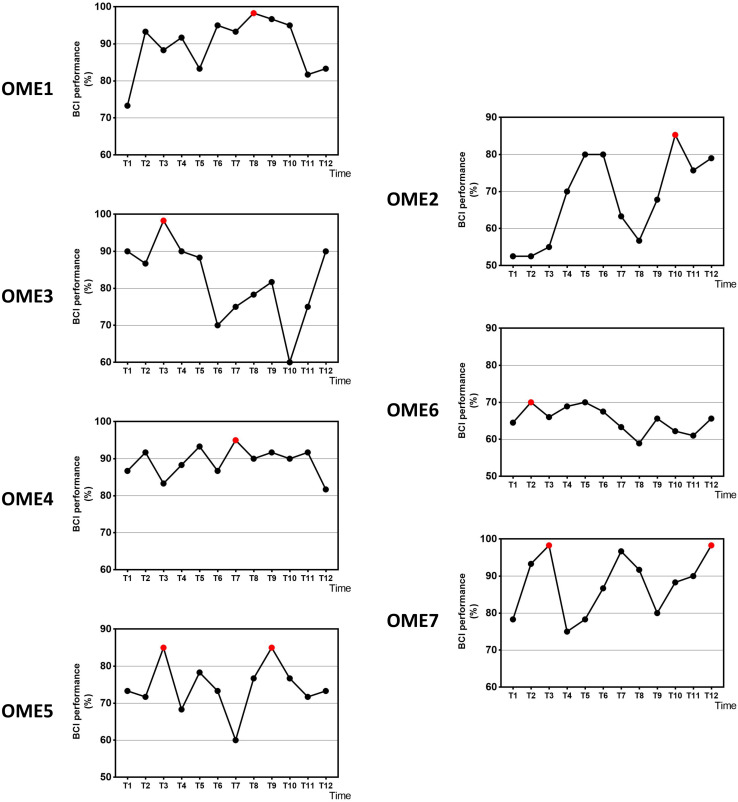
The online BCI performance with 31 channels of the seven subjects during the 12-session BCI training in the BCI group. The red point means the highest value.

Figure 2 shows the online performance of BCI tasks for all subjects. The data were acquired from 31 channels of EEG signals. Three patients (OME1, OME4, and OME7) maintained a level of greater than 70% in BCI performance across the 12 training sessions. Two patients (OME3 and OME5) presented a BCI performance of more than 70% in most of the 12 training sessions. Two patients (OME2 and OME6) showed poor BCI performance during the 12 training sessions.

**FIGURE 3:**
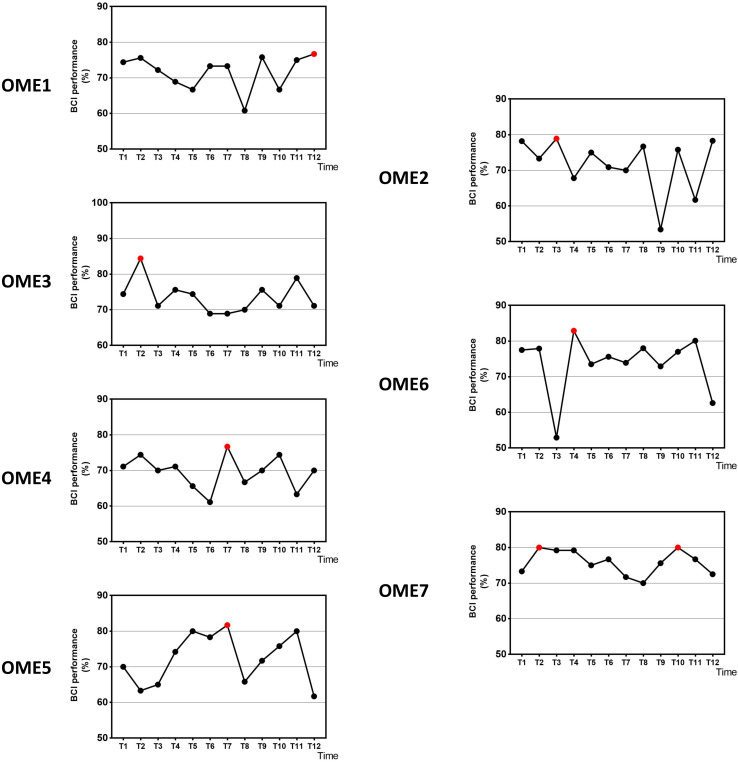
The offline BCI performance with 31 channels of the seven subjects during the 12-session BCI training in the BCI group. The red point means the highest value.

**FIGURE 4:**
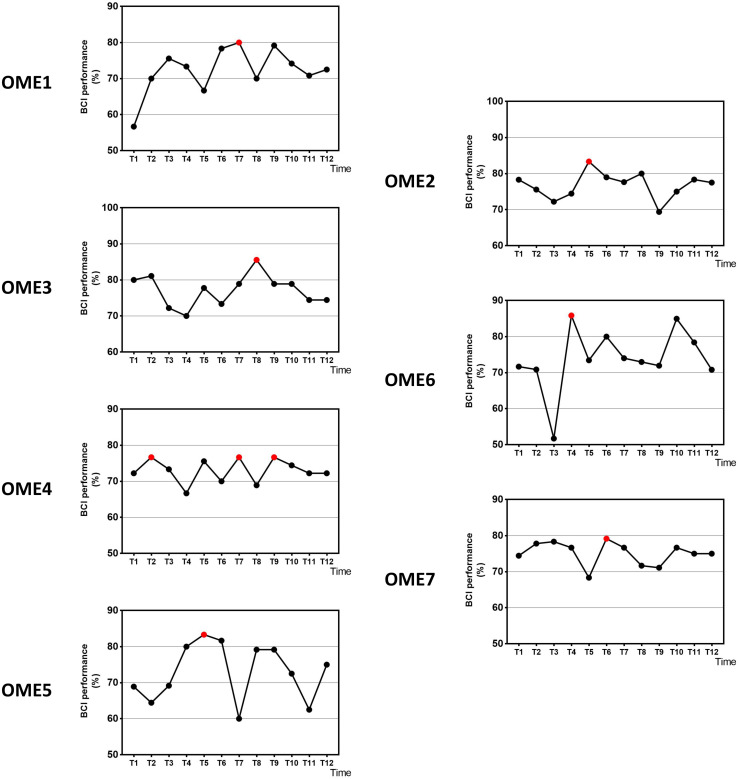
The offline BCI performance with seven channels of the seven subjects during the 12-session BCI training in the BCI group. The red point means the highest value.

Figure 3 shows the offline performance of BCI tasks for all subjects. The data were acquired from 31 channels of EEG signals. Three subjects (OME1, OME4, and OME5) were included into a subgroup, which obtained the highest BCI performance in the last six sessions. Three subjects (OME2, OME6, and OME7) were included into another subgroup, which obtained the highest BCI performance in the first six sessions. Figure 4 shows the offline performance of BCI tasks of for all subjects. The data were acquired from seven channels (FC1, FC2, C3, CZ, C4, CP1, and CP2). Three subjects (OME1, OME3, and OME4) were included into a subgroup, which obtained the highest BCI performance in the last six sessions. Three subjects (OME2, OME6, and OME7) were included into another subgroup, which obtained the highest BCI performance in the first six sessions.

**FIGURE 5:**
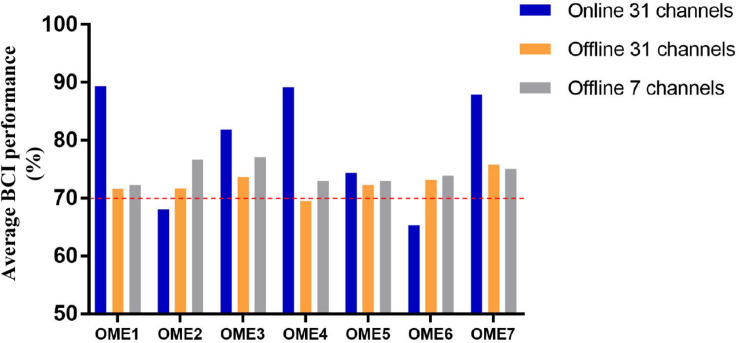
The average BCI performance of 12 sessions with online 31 channels, offline 31 channels, and offline 7 channels of the seven subjects in the BCI group.

Figure 5 shows the average BCI performance of online 31 channels, offline 31 channels, and offline 7 channels. The online accuracies were higher than the offline accuracies in five patients (OME1, OME3, OME4, OME5, and OME7) and lower in two patients (OME2 and OME6). OME2 and OME6 showed average online BCI performance that were lower than 70%. For offline 7 channels and 31 channels analysis, all subjects almost achieved the criterion level of mean accuracy (70%) that rendered the control of BCI application. It was suggested that the pattern of motor attempt could be detected for BCI recognition. However, the mean accuracies used in 7 channels were higher than those used in all 31 channels in six subjects except OME7. It was inferred that the effective features of neural pattern were mainly evoked in the area of motor cortex.

### Rehabilitation Outcomes

**FIGURE 6:**
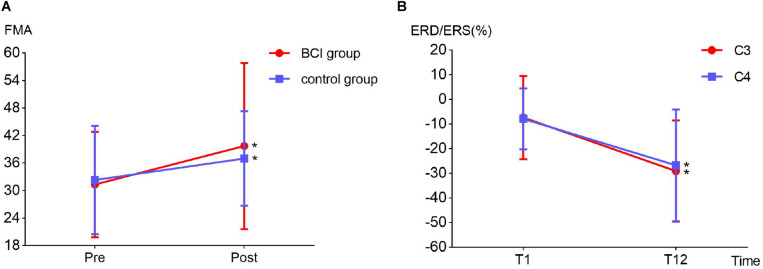
**(A)** Fugl–Meyer assessment improvement between BCI group (12.77%, *p* = 0.032) and the control group (7.14%, *p* = 0.048) before (T1) and after (T12) the intervention. **(B)** Event-related desynchronization change in the ipsilesional (channel C3, *p* = 0.032) and contralesional (channel C4, *p* = 0.029) hemispheres before (T1) and after (T12) the intervention in the BCI group.

Two-way repeated-measures ANOVA results for the FMA-UE showed no significant time and group interaction [*F*_(1, 6)_ = 1.209, *p* = 0.314]. The main effect analysis showed that time had a significant effect on FMA-UE [*F*_(1, 6)_ = 12.115, *p* = 0.013], but no significant effect on FMA-UE for group [*F*_(1, 6)_ = 0.009, *p* = 0.926]. Further analysis showed that, after the 1 month intervention, the FMA-UE of the BCI group and that of the control group were both significantly improved. The percentage of improvement of the BCI group (12.77%, *p* = 0.032) was more than that of the control group (7.14%, *p* = 0.048) before and after intervention. The results are shown in Figure 6A. Four (OME1, OME3, OME4, and OME5) of the seven patients (57.1%) in the BCI group obtained more than five scores in FMA-UE improvement, and only two of the seven patients (28.6%) in the control group had improvement of more than five scores in FMA-UE. The detailed improvement of every individual is shown in Table 2.

**TABLE 2:** Fugl–Meyer assessment scores change between the BCI group and the control group.

FMA-UE	Pre-intervention	Post-intervention	Improvement
**BCI group**			
OME1	36	45	9
OME2	30	32	2
OME3	50	65	15
OME4	37	60	23
OME5	28	34	6
OME6	25	28	3
OME7	13	14	1
**Control group**			
CG1	19	34	15
CG2	28	31	3
CG3	29	36	7
CG4	26	28	2
CG5	28	30	2
CG6	42	42	0
CG7	54	58	4
			

### ERD and Topography Changes

The detailed ERD changes (in T1 and T12 intervention) of every individual are shown in Table 3. It showed that the averaged ERD of ipsilesional (channel C3) and the contralesional (channel C4) hemisphere of seven subjects became stronger after the 12-session BCI training. Two-way repeated-measures ANOVA results for the ERD of channels C3 and C4 showed no significant time and channel interaction [*F*_(1, 6)_ = 0.319, *p* = 0.593]. The main effect analysis showed that time had a significant effect on ERD [*F*_(1, 6)_ = 8.927, *p* = 0.024], but no significant effect on ERD for channel [*F*_(1, 6)_ = 0.188, *p* = 0.680]. Further analysis showed that ERD of both channels C3 (*p* = 0.032) and C4 (*p* = 0.029) became significantly stronger after the 12-session BCI interventions. The results are shown in Figure 6B.

**TABLE 3:** Event-related desynchronization values before (T1) and after (T12) the BCI intervention of the ipsilesional hemisphere and contralesional hemisphere in the BCI group.

ERD	T1		T12	
		
BCI group	Ipsilesional	Contralesional	Ipsilesional	Contralesional
OME1	0.103	0.018	–0.554	–0.505
OME2	0.055	–0.007	–0.126	–0.111
OME3	–0.008	–0.059	–0.242	–0.120
OME4	–0.392	–0.314	–0.607	–0.587
OME5	–0.092	0.024	–0.155	–0.106
OME6	–0.182	–0.176	–0.245	–0.416
OME7	–0.002	–0.040	–0.110	–0.034
Mean	–0.074	–0.079	–0.291	–0.268

**FIGURE 7:**
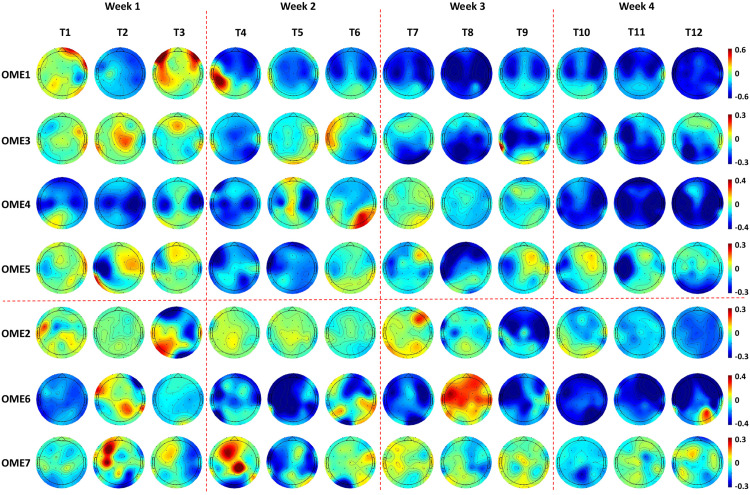
Topographies of 1.4–1.6 s after the task onset of the seven subjects during the 12-session BCI training in the BCI group. Two patients (OME1 and OME3) presented increasingly stronger ERD in the bilateral hemispheres as the training sessions went on. Two patients (OME4 and OME5) presented continuously strong activations (ERD) in the left ipsilesional hemisphere across most of the training sessions. Two patients (OME2 and OME7) presented weak or even no activation in the right ipsilesional hemisphere in more than six training sessions. One patient (OME6) presented ERD or ERS in the bilateral hemispheres in the 12 training sessions.

Topographies in Figure 7 show that OME1 and OME3 presented with increasingly stronger ERD, especially in the ipsilesional sensorimotor cortex (around channel C3) as the training sessions went on. OME4 and OME5 presented continuously activations (ERD) in the left ipsilesional hemisphere across most of the training sessions. OME2 and OME7, who had injury in the right contralesional hemisphere (around channel C4), showed limited or even no activation in the sensorimotor cortex in more than six training sessions. OME6 presented ERD or ERS in the bilateral hemispheres in the 12 training sessions.

## Discussion

Our study obtained more motor function improvement after 12-session BCI intervention in the BCI group than in the control group. Patients with good recovery showed relatively higher online BCI performance, which were greater than 70%. And they showed a continuous improvement in offline BCI performance and obtained a highest value in the last six sessions of interventions during BCI training, whereas patients with poor recovery reached a platform in the first six sessions of interventions and did not improve any more or even show a decrease. Stronger ERD appeared along with motor recovery. Topographies showed that the locations of ERD transferred to be focused on the sensorimotor cortex after BCI intervention.

Brain–computer interface performance was an important parameter of BCI system. Higher BCI accuracies have been correlated with larger excitability in healthy people (Niazi et al., 2012) and better motor recovery in hemiplegic patients (Biasiucci et al., 2018). During BCI training, it would change with the sessions increasing (Li et al., 2014). Many BCI researchers were studying the effects of BCI performance on the motor recovery of stroke patients. Feedback was an essential part of BCI system, and it could promote brain plasticity. Frolov et al. (2017) demonstrated motor function improvements in both subacute and chronic stroke patients with exoskeleton feedback of BCI intervention compared to a control group. Ang et al. (2014) reported that 136 times of BCI exoskeleton feedback could obtain similar recovery effects as 1,040 times of traditional robotic training, which has shown the value of BCI feedback. Higher BCI performance could increase the correct numbers of feedback, which was good for the stroke patients to get motor recovery.

It has been reported in several BCI studies that BCI performance were significantly associated with the improvement of upper limb motor function (Li et al., 2014) or even related to the rehabilitation efficacy of the stroke patients (Bundy et al., 2017; Frolov et al., 2017). In the current study, the rehabilitation process was observed by BCI performance. The group (OME1, OME3, and OME4) that had more improvements in FMA-UE got the highest precision of BCI control in last six sessions of BCI training. While the other group with poor motor recovery (OME2, OME6, and OME7) got it in first six sessions (Figure 4). Among the three patients, OME2 presented poor online BCI performance across the 12 training sessions, with an average accuracy of less than 70%. It was implied that rehabilitation effect was correlated with the performance of BCI control. The steadily rising BCI performance suggested a good learning effect, which might be the source of enhance brain plasticity and motor recovery. The continuous improvements of patients could be reflected by the raise of BCI classification accuracy. And the patients of weak recovery performed more poorly after first several BCI training sessions. It was meaningful for clinical evaluation of recovery effects according to the continuous results of BCI performance.

The power change of EEG during motor task of BCI training was a symbol of brain function. Event-related desynchronization represented the cortical activation state, and stronger ERD suggested better brain function and brain plasticity (Ono et al., 2015). After the 12-session BCI intervention, ERD became significantly stronger in the 12th session compared to the first session. Two patients (OME1 and OME3) obtained obviously strong ERD in the third and fourth weeks. Moreover, the ipsilesional hemisphere presented more power decrease than the contralesional hemisphere did (Table 3). This was consistent with the study in healthy subjects that motor task was mainly activating the contralateral hemisphere (Pfurtscheller, 1999; Wolpaw, 2000). In functional MRI studies, activation on the ipsilateral side was also reported in stroke recovery (Erik Ween, 2008). This also suggested that BCI could facilitate ipsilesional cortical activations, and these activations might lead to motor recovery in subacute stroke patients. Interestingly, one patient (OME7) presented a relatively acceptable online BCI performance (mostly ranking from 70 to 80%). However, he showed poor activations in the right ipsilesional hemisphere, especially around the C4 channel. Thus, OME7 showed poor motor recovery. In addition, OME2 showed poor online BCI performance along with poor activations (ERD) in bilateral hemispheres, especially in the right ipsilesional hemisphere, in most of the 12 training sessions. These might also show poor motor recovery. It suggested that both BCI performance and sensorimotor rhythm (ERD) were important in reflecting motor recovery in subacute stroke patients during BCI training.

The locations of ERD changed as the intervention sessions increased, and specific characteristics were shown by topographies. A patient (OME1), who showed activations in the contralesional hemisphere in the first week, had focal ERD in the ipsilesional hemisphere after several BCI sessions. It was reasonable because the sensorimotor areas should be mostly involved in motor-related tasks (Li et al., 2014; Wang et al., 2016). Patients, who presented with extensive activations at the very start, became focused on the sensorimotor cortex after BCI training. This suggested that as the BCI intervention continuously proceeded, neural plasticity might enhance with stronger ERD in the sensorimotor cortex, which might lead to better motor recovery. Additionally, a patient (OME6) who presented with strong ERD but poor motor recovery could be at a subclinical efficacy. He also presented poor online BCI performance of less than 70%. Actually, he got an improvement of 3 in FMA-UE, which was lower than did the other patients with good motor recovery. He might have a slower improvement than others but might be further improved with more training.

The limitations of our study included a small sample size and no EEG data collected from the control group. This led to a lack of EEG comparison between groups and a more moderate conclusion of the clinical efficacy of BCI intervention. Based on this pilot study, a further study with a larger sample size is needed to contribute a stronger and clearer result.

## Conclusion

This study explored the longitudinal sensorimotor rhythm changes in subacute stroke patients after 1 month BCI training with exoskeleton feedback. Clinical improvement was found in FMA-UE scores. Brain–computer interface performance was a good index to evaluate the clinical efficacy during the long-term BCI intervention. Patients who presented increasingly stronger or continuously strong activations (ERD) may obtain better motor recovery.

## Data Availability Statement

The raw data supporting the conclusions of this article will be made available by the authors, without undue reservation.

## Ethics Statement

The studies involving human participants were reviewed and approved by the Ethics Committee of Huashan Hospital. The patients/participants provided their written informed consent to participate in this study. Written informed consent was obtained from the individuals for the publication of any potentially identifiable images or data included in this article.

## Author Contributions

SC and XS designed and performed the study. SC and LC organized the data, performed the data analysis, and wrote the manuscript. LC, XS, HW, LD, S-HW, and JJ reviewed and edited the manuscript. All authors read and approved the submitted manuscript.

## Conflict of Interest

The authors declare that the research was conducted in the absence of any commercial or financial relationships that could be construed as a potential conflict of interest.
